# In situ laboratory for plastic degradation in the Red Sea

**DOI:** 10.1038/s41598-022-15310-7

**Published:** 2022-07-13

**Authors:** Franz Brümmer, Uwe Schnepf, Julia Resch, Raouf Jemmali, Rahma Abdi, Hesham Mostafa Kamel, Christian Bonten, Ralph-Walter Müller

**Affiliations:** 1 Institute of Biomaterials and Biomolecular Systems, Research Unit Biodiversity and Scientific Diving, University of Stuttgart, Pfaffenwaldring 57, 70569 Stuttgart, Germany; 2 Institut für Kunststofftechnik IKT, University of Stuttgart, Pfaffenwaldring 32, 70569 Stuttgart, Germany; 3 Scientific Diving Group University Stuttgart (WiTUS), University of Stuttgart, Pfaffenwaldring 57, 70569 Stuttgart, Germany; 4 Institute for Structure and Design DE, German Aerospace Institute (DLR), Pfaffenwaldring 38 - 40, 70569 Stuttgart, Germany; 5 Beluga Egypt, 32 Bahaa Eldin Elghatwary St, Semoha 12th floor apartment 1203, Alexandria, Egypt

**Keywords:** Environmental impact, Materials science, Marine chemistry, Environmental impact, Environmental monitoring

## Abstract

Degradation and fragmentation of plastics in the environment are still poorly understood. This is partly caused by the lack of long-term studies and methods that determine weathering duration. We here present a novel study object that preserves information on plastic age: microplastic (MP) resin pellets from the wreck of the SS Hamada, a ship that foundered twenty-nine years ago at the coast of Wadi el Gemal national park, Egypt. Its sinking date enabled us to precisely determine how long MP rested in the wreck and a nearby beach, on which part of the load was washed off. Pellets from both sampling sites were analyzed by microscopy, X-ray tomography, spectroscopy, calorimetry, gel permeation chromatography, and rheology. Most pellets were made of low-density polyethylene, but a minor proportion also consisted of high-density polyethylene. MP from inside the wreck showed no signs of degradation compared to pristine reference samples. Contrary, beached plastics exhibited changes on all structural levels, which sometimes caused fragmentation. These findings provide further evidence that plastic degradation under saltwater conditions is comparatively slow, whereas UV radiation and high temperatures on beaches are major drivers of that process. Future long-term studies should focus on underlying mechanisms and timescales of plastic degradation.

## Introduction

The usage of plastics has led to numerous improvements in everyday life^1,2^, but has recently been recognized as an environmental thread on a global scale that exceeds planetary boundaries due to high production rates and uncontrolled littering^3^. Plastics have been discarded into the environment for at least five decades^4,5^. As a consequence, plastics can be found everywhere^6^, with at least five billion plastic pieces that have entered the oceans, a major sink for plastic debris^7^. It was also reported that about 275 million metric tons of plastic waste was generated in 192 coastal countries in 2010, with 4.8 to 12.7 million megaton entering the ocean^8^.

Recently, a special focus was set on plastics in the size range from 1 µm to 5 mm, which are called microplastics (MP)^9–11^. There are two types of MP: primary and secondary MP. Primary MP is deliberately produced in this size range for many purposes, e.g., cosmetic and hygiene products or resin pellets for industrial usage^12,13^. Pellet loss during production, transport, storage or waste management can act as an important input pathway for primary MP into marine habitats^14,15^. Secondary MP originates from the fragmentation of larger plastic pieces^12^.

Regardless of its formation, MP can undoubtedly induce adverse effects on marine wildlife and ecosystem services, down from the cellular level of a single individual up to an acceleration of climate changes^16,17^. For instance, MP act as both, sinks and sources of potentially toxic substances, i.e., persistent organic pollutants, metals, additives, plasticizers and antibiotics^18–22^. This chemically complex cocktail might be released at a constant, yet increasing rate due to plastic fragmentation, resulting in a so-called global plastic toxicity debt^23^. In addition, MP has the potential to accumulate in marine food webs as a consequence of trophic transfer^24,25^. Once ingested by organisms, MP can cause physical damage of tissue, e.g., intestinal obstructions, inflammation processes, and might also influence animal behavior in a negative manner^26^. Nanoplastics, which are defined as plastic particles with a size between 1 nm and 1 µm, are even able to pass biological membranes and, thus, can directly interact with genetic material and cellular organelles^27^. Ultimately, this could change population structure and abundance of certain species. On a global scale, plastics release CO_2_, methane and an array of other greenhouse gases at each stage of their life cycle, thereby, contributing to climate change in a meaningful proportion^28^. For example, in 2015, plastics alone were responsible for 4.5% of the world´s greenhouse gas emissions^17^. In addition to this, MP may disturb the biogeochemical processes with which plankton capture CO_2_ at the sea surface and sequester carbon in the deep oceans, although this is still poorly understood ^29^. As a consequence of these negative ecological impacts, marine littering of plastic debris is considered to be part of a global crisis^30^.

The aforementioned long lasting effects of MP are partly a consequence of their high persistence in natural systems which are estimated to last from hundreds to thousands of years^31^. For instance, in marine environments the halve life of a plastic bag made of low-density polyethylene (LDPE) with an average specific surface degradation rate of 15 µm per year is estimated to be in the range between 1.4 and over 2500 years^32^. This is slow compared to other polymers, according to a three-level machine learning classification that takes into account a bunch of physical and molecular characteristics^33^. Besides the very properties of MP, another important set of factors determining the extent of plastic degradation are environmental conditions themselves. These include thermo-oxidative stress, shear forces by water movement, humidity, biological activity from upgrowing organisms, and photo degradation by UV irradiation^32,34,35^. The latter is considered to usually initiate degradation in marine environments^36^. With ongoing exposure, the surface of plastic tends to crack and, finally, surface ablation as well as fragmentation leads to the release of secondary MP^37^. Although some authors studied plastic degradation under marine conditions for several years, only very few long-term examinations have been undertaken so far to our knowledge^38–40^. Also, due to the lack of reliable analytical methods age and weathering time cannot be estimated directly from plastics in the environment^37^. Both parameters depend on varying exposure conditions at changing sites and this information is usually not available to researchers.

**Figure 1:**
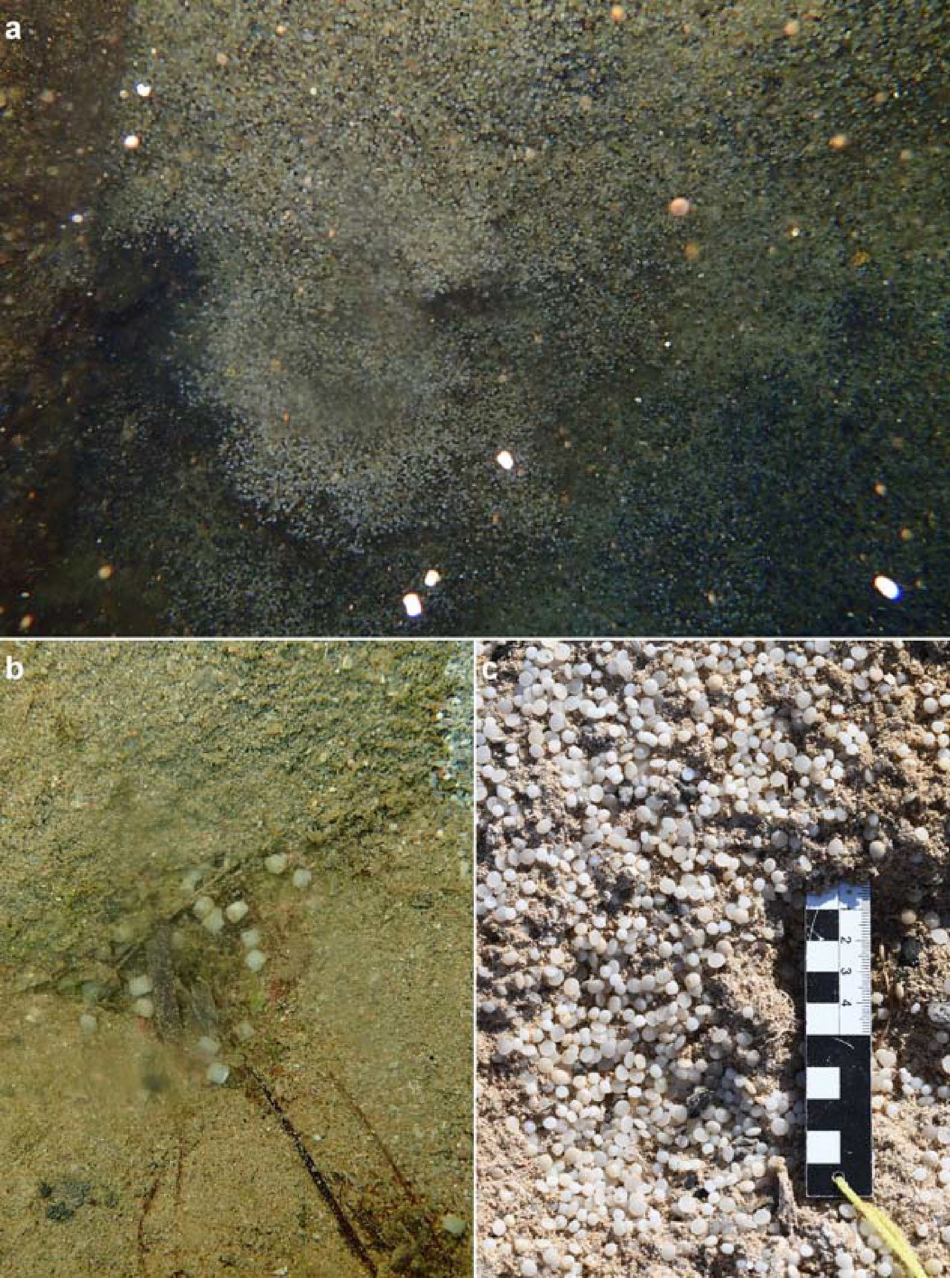
Fate of MP resin pellets from a beach in the Wadi el Gemal national park, Egypt, and the SS Hamada wreck in the Red Sea. (**a**) In the dark waters of the ship wreck, thousands of pellets floating up and down. (**b**) While some sacks fell off the ship and released fouled plastics to the surrounding sea floor after they have been torn open, other parts of the load (**c**) floated to the sea surface, from which they drifted to a nearby beach, which act as a highly covered plastic sink.

One such exceptional case is linked to the foundering of the bulk carrier SS Hamada near Abu Ghosun in the Wadi el Gemal national park, Egypt, around twenty-nine years ago on 29th June 1993^41,42^. On its last cargo transport, the ship – 65 m in length and 11 m in width—was loaded with primary MP resin pellets. Today, the SS Hamada wreck lies on its starboard side in two sections, partly with keel up at the sea ground, and is a popular an easily accessible diving spot in around 6 to 18 m’ depth. Its cargo holds are still filled with MP resin pellets (Fig. 1a), but many were released as a result of foundering. Many MP floated to an adjacent beach (Fig. 1c). In comparison to this, fewer MP ended up at the sea floor (Fig. 1b). They originated from sacks, that fell off the SS Hamada during the foundering and have been torn open later. Because the included MP resin pellets were overgrown by fouling organisms in the meantime, they became negatively buoyant and, thus, remained at the sea floor^34^.

We propose that MP from the SS Hamada wreck is an inimitable case for long-term studying of plastic degradation in the environment. But to which extent did the MP resin pellets degrade so far? To answer this question, we collected free floating MP resin pellets from inside the SS Hamada wreck and MP resin pellets from the beach directly in front of the sinking place to subject them to a comparative analysis. For this, a sophisticated combination of imaging methods, particle measurement, infrared spectroscopy, thermal analysis, biochemical approaches, and rheology was applied. As beaches provide optimal conditions for plastic degradation^43^, we created the hypothesis that MP resin pellets from the beach to weathered faster than those under dark and colder conditions inside the SS Hamada wreck on all structural levels.

## Results

The SS Hamada ship wreck acts like an in situ laboratory for plastic degradation due to the exactly dated MP resin pellets released by it. Plastics from inside this wreck and the nearby beach were sampled to scrutinize possible differences in the extent of plastic degradation due tod dissimilar environmental conditions.

### Polymer type

**Figure 2:**
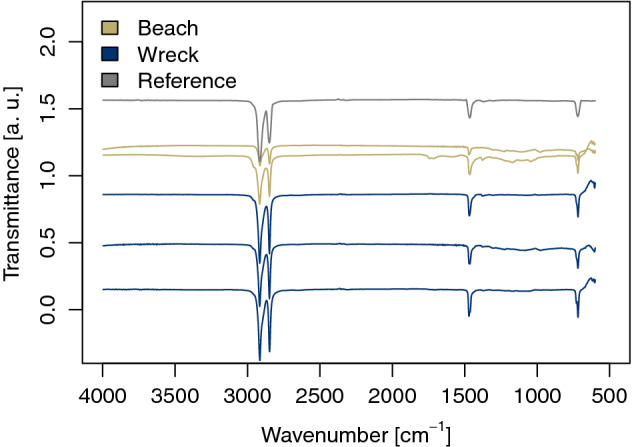
Fourier-transform infrared spectra measured on the inside of MP resin pellets from a beach in the Wadi el Gemal national park, Egypt, and the SS Hamada wreck in the Red Sea. By comparison with a reference database, most MP were identified as LDPE. Their carbonyl index (1810–1550 cm^-1^) and the fingerprint area (< 1500 cm^-1^) revealed chemical modifications only in the case of MP from the beach.

Fourier-transform infrared spectroscopy (FTIR) was used to determine the polymer types of MP resin pellets from the SS Hamada. They could be identified as LDPE (Fig. 2). Note that a very small proportion of high-density polyethylene (HDPE) was detected, too. Average Pearson´s r indicated a lower match for beached MP resin pellets as compared to plastics from the ship wreck (0.77 and 0.95, respectively).

Besides polymer identity, possible degradation features can also be analyzed by FTIR. Differences between sample sites were identified in the fingerprint area (< 1500 cm^-1^). While the wreck spectra showed no meaningful deviation from the reference in the fingerprint area, the beach samples exhibited considerably stronger overlaps and stretching vibrations, which are apparent in the form of broad peaks (Fig. 2). This is a sign that their structure was chemically altered. In particular, ranges of higher wave numbers indicated the presence of certain functional groups. Note that there were no meaningful changes in the spectral behavior of MPs from both sample sites in the range of 1500–4000ؘ cm^-1^ compared to a database reference except for one beached MP that had an absorption band at 1800 cm^-1^ (Fig. 2). This specific pattern is an indication for carbonyl groups formed during aging.

### Particle size and shape distributions

Both MP particle collectives had comparable particle size and shape descriptor distributions (Supplementary Table S2 online).

MP resin pellets found on the beach had a median size of 3.7 mm with an IQR of 0.2 mm, while those from the wreck had a median size of 3.8 mm and an IQR of 0.3 mm (Supplementary Fig. S2 online).

**Figure 3:**
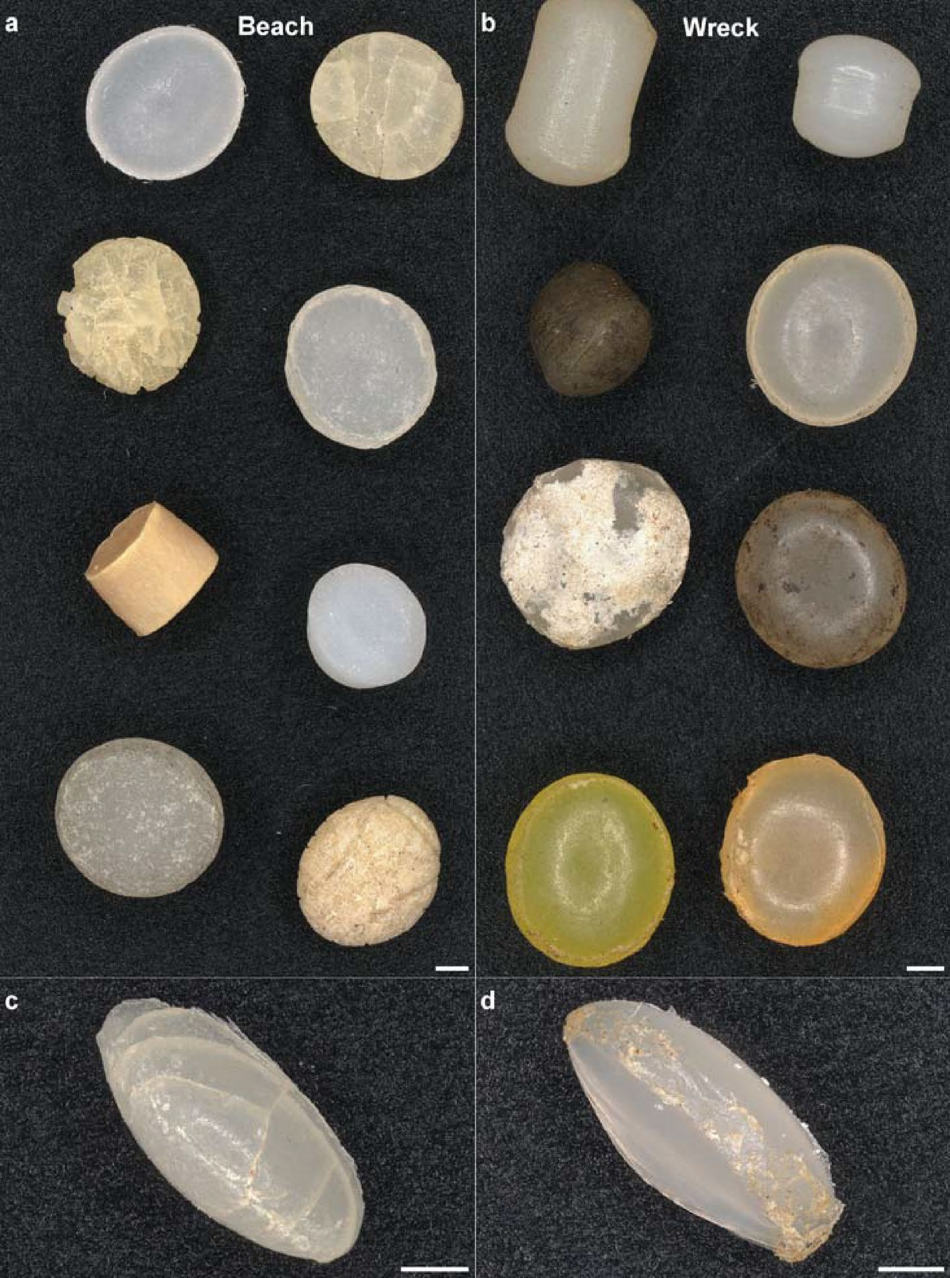
Light microscopic images of MP resin pellets from a beach in the Wadi el Gemal national park, Egypt, and the SS Hamada wreck in the Red Sea. Representative overview of different particle shapes and colors of MP from (**a**) the beach and (**b**) the SS Hamada wreck. The proportion of this properties varied within the sample, in which beige, white, and grey disk-like pellets were more frequent than yellow, black, red, or cylindrical particles (c.f. Supplementary Fig. S2 online and Supplementary Fig. S3 online). A complex network of cracks was only present on beached MP. Side views of particle surface revealed a lower level of abrasion for (**c**) MP from the beach compared to (**d**) the wreck. Scale bars: 1 mm.

The majority of MP was classified as disk-like pellets, although a minor amount of cylindrical particles was also present (Fig. 3a–b, Supplementary Fig. S2 online, Supplementary Table S2 online). Values for isoperimetric shape factor *f*_*1*_ (Eq. 1) were close to one, with an average of 0.83 ± 0.06 (mean ± s.d.) and 0.82 ± 0.1 for MP from the beach and the wreck, respectively (Supplementary Fig. S2 online). However, a small proportion of analyzed pellets, i.e., cylinders, had values of *f*_*1*_ < 0.60. Elongation showed a left skewed distribution for all MP exhibiting average values of 0.16 ± 0.05 (beach) and 0.18 ± 0.10 (wreck) (Supplementary Fig. S2 online). Flatness followed a bimodal distribution with a mean of 0.61 ± 0.19 for beached MP resin pellets and 0.56 ± 0.27 for particles from the SS Hamada wreck (Supplementary Fig. S2 online). Low values of flatness were typical for disks, while a high flatness was exclusively detected for cylindrical MP.

### Color

Contrary to the particle size and shape descriptor distributions, proportions of MP color were dissimilar for the two sample sites (Supplementary Fig. S3 online). On the beach and inside the wreck, white (63% and 22%, respectively) and beige (31% and 41%, respectively) were the predominant colors, followed by a minor amount of grey (2 and 12%, respectively) and yellow MP (13% and 14%, respectively). Note that beached MP showed only a weak yellow on the surface, while particles from the wreck exhibited a deep yellow in all layers. In addition, MP resin pellets from the SS Hamada wreck were also red or black (both 1%), whereas these colors were absent in the beach subsample.

### Morphology

Imaging methods were applied to qualitatively assess morphological signs of MP degradation. In general, MP resin pellets from the beach showed typical signs of degradation, while they were absent in case of particles from the SS Hamada wreck.

**Figure 4:**
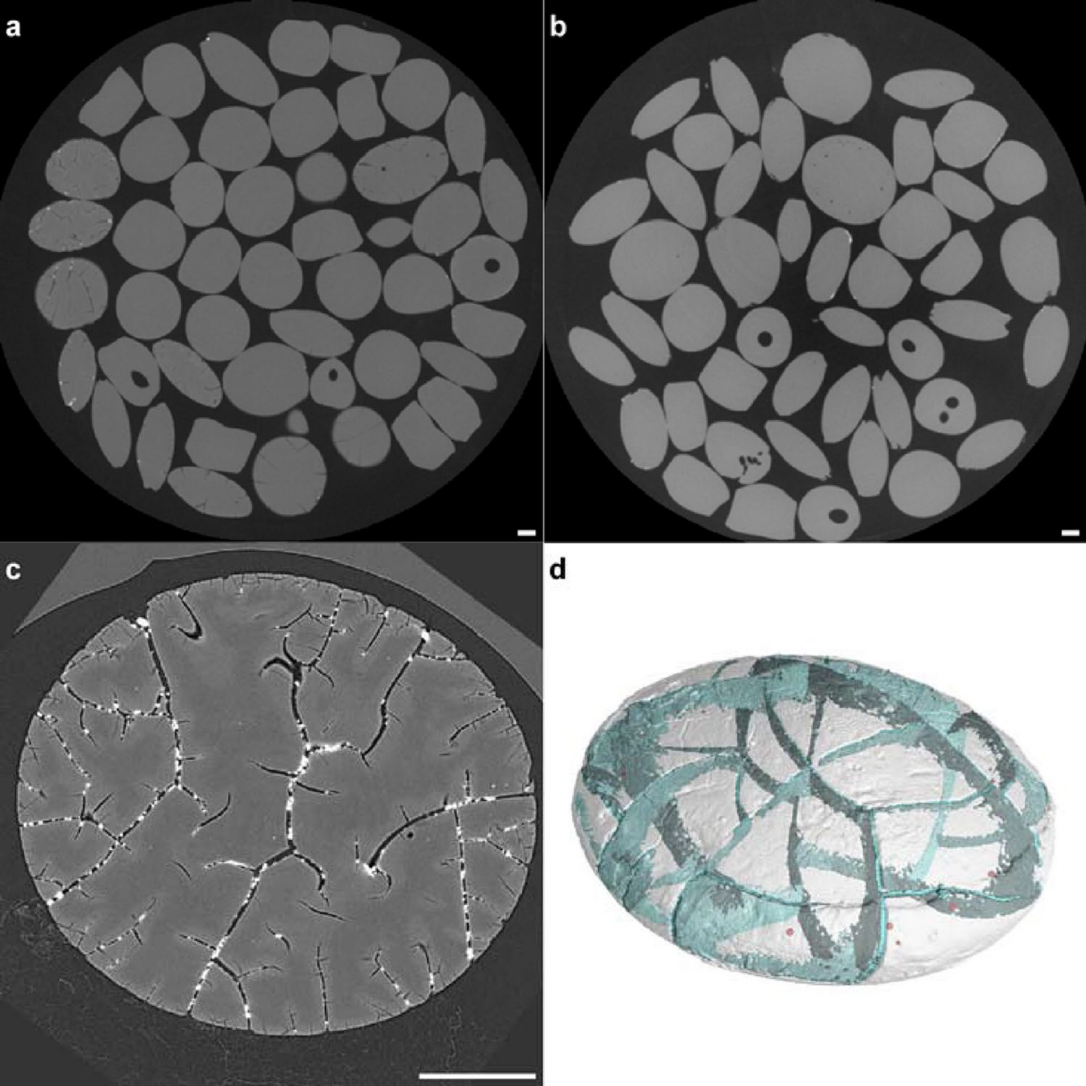
Slices of µCT scanned MP resin pellets from a nearby beach in the Wadi el Gemal national park, Egypt, and the SS Hamada wreck in the Red Sea. Overviews of different MP from (**a**) the beach and (**b**) from SS Hamada wreck revealed several cavities that are most likely a result of production conditions. (**c**) Pellets from the beach often showed white spots that indicate the presence of minerals inside distinctive cracks. (**d**) 3D reconstructions of µCT slices exhibited that cracks (blue) formed a complex network which reached the inner part of beached MP. Further crack propagation will most likely lead to fragmentation of pellets into even smaller MP. Red dots indicate closed pores inside the pellet. Scale bars: 1 mm.

No biofouling occurred in case of MP from the beach, but the surface was frequently covered with a dense network of scales and cracks (Fig. 3a, Supplementary Fig. S4 online). With ongoing crack propagation, cracks tended towards the center of pellets and developed several bifurcations (Fig. 4a,c,d). Also, some cracks united with other cracks. White shades were detected inside the cracks, indicating that the crack network itself is filled with a notable amount of minerals (Fig. 4c). Noteworthy, the sides of MP particles were smooth, and no shredded areas were visible (Fig. 3c).

On the contrary, MP from the wreck showed a virgin like surface (Fig. 3b). However, glazed surfaces were observed on a few particles due to the presence of chalky appearances (Fig. 3b). Likewise, other attachments were visible mainly on the sides of the pellets and can be attributed to biofilm formation (Fig. 3d). Additionally, the sides of MP sometimes showed shredded areas (Fig. 3d).

On the inside of some pellets from both sample sites, cavities were visible (Fig. 4a–b). They could be attributed to air inclusions caused by the manufacturing process.

### Mass

In case of particle mass, the mean difference between sample was not considered to be meaningful (-0.146 mg, 95% CI [-1.515 mg, 1.223 mg], Supplementary Fig. S6 online). MP from the beach on average weighed 25.313 ± 0.484 mg, while those from the wreck had a mean mass of 25.459 ± 0.498 mg (mean ± s.e.m., Welch’s two-tailed t test: t(198) = -0.21, *p* = 0.834).

### Differential scanning calorimetry

**Figure 5:**
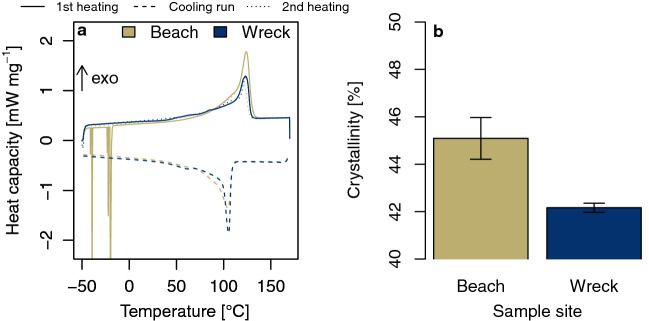
Thermal DSC analysis of MP resin pellets from a beach in the Wadi el Gemal national park, Egypt, and the SS Hamada wreck in the Red Sea. (**a**) Average of ten DSC curves for each sample site. The higher the melting peak in the heating runs, the higher the degree of crystallization. (**b**) Crystallinity of beached MP was higher than for pellets from the wreck. Values are given as mean ± s.e.m. for n = 10 replicates.

Differential scanning calorimetry was applied to assess thermal behavior of MP collected at both sample sites. The presence of co-polymers or blends could be excluded as only single and clearly distinguishable peaks were detected (Fig. 5a). A glass transition region in the first heating cycle and polymer recrystallization in the cooling run were clearly visible (Fig. 5a). Both are typical for semi-crystalline thermoplastics like LDPE.

During the second heating run, a shift of the glass transition region was found. This was probably caused by the evaporation of plasticizers. More defined melting peaks in the second heating cycle suggest an increased purity of the polymer. The reduced area under the peaks suggests a decrease of mass. Crystallinity was higher for MP resin pellets from the beach than for those from the wreck, with a mean difference of 2.93% (95% CI [0.92%, 4.94%], Welch’s two-tailed t test: t(10) = 3.252, *p* = 0.009, Fig. 5b).

### Gel permeation chromatography

Gel permeation chromatography (GPC) enables the separation of macromolecules according to their size. From this, branch length of monomers can be described either by average molar mass weighed by number (M_n_) or by mass (M_w_). As no pristine material was available, data was compared with previously published results^44^.

Note that MP from the beach could not be fully dissolved because of cross-linked polymer chains and, hence, the measurement could not be used for a quantitative analysis. Even after extending solution from 2 h at 150 °C to 120 h under standard conditions, MP from the beach could not be dissolved in the 1,2,4-trichlorbenzene eluent. Instead, beached MP have swollen, and they were clearly visible until the end of the dissolution test.

**Figure 6:**
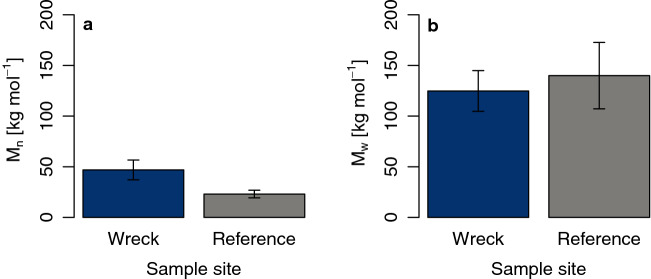
GPC results to study plastic degradation on a molecular level. We determined (**a**) number average molar mass (M_n_) and (**b**) mass average molar mass (M_w_) of MP resin pellets from the SS Hamada wreck in the Red Sea. No data for MP resin pellets from the beach is shown here as it was impossible to dissolve particles. Reference data for LDPE was obtained from the literature^44^. Values are given as mean ± s.e.m. for n = 5 replicates in case of MP from the SS Hamada wreck and n = 6 for reference data.

For MP from the SS Hamada wreck, M_n_ was smaller than M_w_ with a ratio of approximately 1:3 (46.85 ± 9.78 kg mol-^1^ and 124.80 ± 20.06 kg mol-^1^, mean ± s.e.m., respectively; Fig. 6). Compared to the reference, M_n_ was -23.85 kg mol-^1^ smaller for MP from the wreck (95% CI [-50.50 kg mol-^1^_,_ 2.80 kg mol-^1^], Welch´s two-tailed t test: t(5) = -2.277, *p* = 0.070), while M_w_ was 15.20 kg mol-^1^ larger (95% CI [-73.36 kg mol-^1^, 103.76 kg mol-^1^], Welch´s two-tailed t test: t(8) = 0.395, *p* = 0.703).

### Rheology

As molecular weight distribution and level of long-chain branching slightly changed during weathering of plastics under different environmental conditions, this might have affected rheological measures, too.

**Figure 7:**
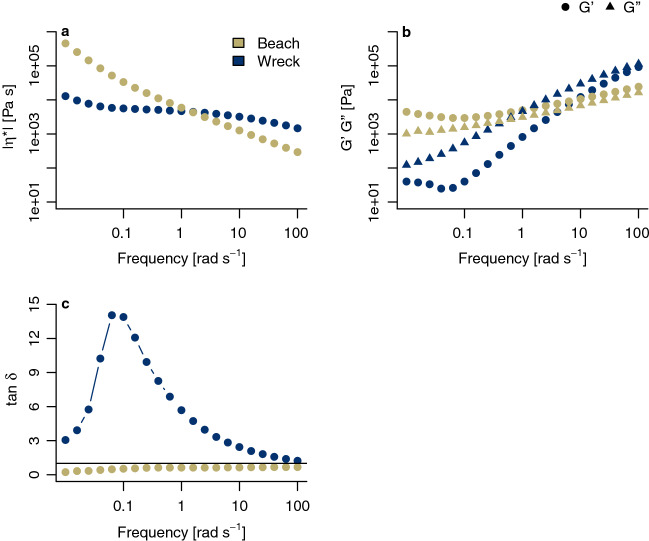
Rheological properties of MP resin pellets from a beach in the Wadi el Gemal national park, Egypt, and the SS Hamada wreck in the Red Sea. MP from the wreck showed a behavior typical for pristine PE, while beached pellets exhibited a more complex microstructure. (**a**) Log–log-scaled complex viscosity |η*| (Eq. 2). A Newtonian plateau at low frequencies was solely found for MP inside the wreck. (**b**) Storage modulus G´ (circles) and loss modulus G´´ (triangles) on a log–log scale. G´ was constantly higher than G´´ in the case of pellets from the wreck, which is typical for pristine LDPE. For beached MP, the opposite was observed. This indicates a more complex microstructure. (**c**) Loss tangents tan δ was always lower than one (solid line) in the case of beached pellets and higher than one for MP from the wreck. Note the logarithmic scale on the x-axis.

Wreck samples exhibited a S-shaped curve with a Newtonian plateau at low frequencies followed by a shear rate drop (Fig. 7a) Beached MP showed a much higher complex viscosity at low frequencies and a zero-shear viscosity (*η*_*0*_). In contrast, at intermediate to high frequencies, samples from the beach exhibited a five times lower complex viscosity than MP from the SS Hamada wreck (300 Pa and 1500 Pa, respectively). Further, no shear-thinning behavior was observed.

Storage (G′) and loss modulus (G″) as well as loss tangents (tan δ = G’’/G′) are all linear viscoelastic functions, which are more sensitive to changes in the length and height of the cross-linking of PE chain. MP from the SS Hamada wreck showed a predominant viscous behavior (G’’ > G′) and values of tan δ higher than one (Fig. 7b and c). In addition, at high frequencies, a crossover point between G′ and G’’ with a tan δ value of one could be detected. In contrast, the curves of beached MP were clearly flatter, and G’ was consistently higher than G’’. Compared to MP from the wreck, both G’ and G’’ had higher values at low frequencies, and lower values at high frequencies. Furthermore, the beach samples showed a tan δ value lower than one and also a minimum at low to intermediate frequencies (Fig. 7c).

### Comparison of sample sites

Overall, degradation of MP resin pellets was considerably more advanced in case of samples from the beach (Supplementary Table S3 online). They exhibited typical signs of plastic degradation from the molecular level up to morphological changes, while almost anything of this was absent for particles from inside the SS Hamada wreck. Notably, beach samples showed cross-linking of chain branches, higher crystallinity, a set of rheological properties untypical for pristine LDPE, a much higher number of surface cracks resulting in a complex crack network, and finally a higher extent of fragmentation.

## Discussion

To conduct comparative long-term analyses on plastic degradation with plastics of known age, the MP resin pellets from the SS Hamada are another great study object. Here, date of sinking and, consequently, the timepoint at which MP entered the marine environment can be determined to a day. Furthermore, the weathering history is well documented for MP inside the wreck. This information remains fragmentary in the case of beached pellets until novel methods approch^38^. Nonetheless, the PE-MP load of the SS Hamada provides precise and meaningful data on environmental degradation of plastics, that other investigations lack thus far. The most prominent of these cases are LEGO® bricks, but their age can only be bounded by a span of a whole decade^39^. They are also made of acrylonitrile butadiene styrene, a polymer type that is rarely used in industry. Contrarily, the market share of both LDPE and HDPE sums up to a total of 36.3%, which means that they are the most frequently produced plastics in the world^45^. Hence, the exactly dated MP from the SS Hamada are more representative for a very large proportion of plastics that enter marine environments.

Comparative measurements proofed our hypothesis that beached MP has weathered to a greater extent than those in the dark waters inside the wreck. MP on the beach showed distinct morphological changes that frequently occur in this habitat^46–49^, which indicates that this is a general pattern^36^. Particularly, only beached MP showed some extent of yellowing, either caused by fading of pigments, the decay of stabilizers or the polymer itself^37^. Another typical observation are scales, which acted as seed points for cracks that expanded towards the center of beached MP, leading to the formation of a complex network^46^. This process was accelerated by incorporated sea salt or other biogenic minerals that increase friction forces and alter reaction conditions^39^. As opposed to that, the increased crystallinity of beached MP might have slowed down crack propagation and plastic degradation in general^50,51^. Note that the increase of crystallinity will stagnate at some time, because oxygen cannot penetrate into crystalline phases of semi-crystalline polymers like LDPE^36^. Only amorphous phases can re-arrange in the case, cracks will propagate^37^ until they reach cavities, causing MP fragmentation after application of mechanical force from waves or wildlife^48^. As a consequence, secondary MP < 300 µm with irregular shape and even nanoplastics could be released in high quantities^48^.

Both, nanoplastics and MP can negatively impact marine environments. For instance, the high coverage found on the beach increase the permeability of sediments and reduce heat transfer^52^. This may influence biogeochemical processes, nutrient cycling and temperature profiles in a yet unknown way. Therefore, we encourage beach cleanup-activities to protect the vulnerable ecosystems in the Wadi el Gemal national park.

We mostly observed MP with a smooth form. In addition, no difference in particle size and mass loss was detected after twenty-six years. Weathering also did not affect particle size of LEGO® bricks, which rested four to five decades in marine environments^39^. However, these acrylonitrile butadiene styrene-based plastics showed a median mass loss of 18.3% with a range between 2.88 and 39.80%^39^. This is much higher than the average 0.57% mass loss we measured for LDPE. Note that the average mass loss of MP inside the wreck was chosen as baseline as no relevant mass loss could be expected^51^. Summing up, mass loss is a function of polymer type and time, but does not necessarily influence particle size.

Both, the results obtained for particle size and mass indicate that fragmentation rarely occurred, even on the beach. However, mechanical abrasions on the sides of pellets inside the wreck are a notable source of secondary MP. Such kind of surface degradation might be caused by the high mobility of pellets in the water column^48^.

FTIR confirmed the optical impression that beached MP degraded faster. They showed an altered valence oscillation in the fingerprint area. Surprisingly, both the extent of carbonyl groups and hydroxyl groups did not alter during environmental weathering^36^. This could be explained by a lack of chromophores in PE. Thus, photo-degradation is less relevant compared to other synthetic polymers^35^. Also, photo-oxidation is limited to the top 100 µm^53^, so we cannot exclude that it was not possible to measure reaction products inside the particle. Future studies should therefore carefully apply techniques to remove biofilms from beached MP. Pellets inside the wreck did not show spectral anomalies. In this coastal area, only a neglectable proportion of UV radiation penetrates to 18 m’ depth, so photo-oxidation couldn´t be triggered^36,54^.

The non-altered fingerprint range in the IR spectrum of MP inside the wreck went along with no substantial difference in the molecular weight distribution compared to the reference and, consequently, in its molecular structure. However, there is room for uncertainty, as the very properties were unknown in the case pristine MP from the SS Hamada. MP from the wreck behaved like pristine PE, which is a structure viscous polymer. It showed a Newtonian plateau at low frequencies, a shear thinning behavior, a predominant viscous behavior (G’’ > G′), and values of tan δ higher than one.

In contrast to these unchanged rheological properties of pellets from inside the wreck, beached MP developed a more complex microstructure. On the one hand, those pellets exhibited an increase in long-chain branching and cross-linking phenomena, which was a result of changes in the chemical structure^37^. First, they exhibited no Newtonian plateau and a high complex viscosity at low frequencies, which is characteristic for viscoelastic solids. Second, in preparation for GPC, particles have swollen, and the beach sample could not be dissolved in 1,2,4-trichlorbenzene due to cross-links in molecular chains. The insolubility of beached MP gets confirmed by rheological investigations. Third, the beach samples showed a tan δ value lower than one with a minimum at low to intermediate frequencies, which also indicates improved relative elastic reactions. Summing up, rheology provided sufficient evidence that MP polymer branches lengthened. On the other hand, chain scission co-occurred. Both chain length and molar mass of the polymer chains have been reduced. This is indicated by an increase of complex viscosity |η*| and the corresponding shear thinning behavior. The lower complex viscosity at high frequencies indicates a lower molecular weight of the chains. The results of the investigation of the storage modulus and the loss modulus support this. Note that a release of mono- and oligomers can cause toxic effects^36^.

Although we observed some chain scission, it is unlikely that biodegradation played a substantial role in the decay of MP resin pellets from the SS Hamada. For this, M_n_ must reach values around 0.5 kg mol^-1^ so that microbes can mineralize PE, but MP from the wreck exceeded this limit 100-fold^34^. Hence, fundamental drivers of plastic degradation are abiotic factors, i.e., UV radiation, wind, wave and tide actions^35^.

In conclusion, we provide further evidence that beached MP degrade faster than those in the oceans. The main factors of influence are photo-oxidation and mechanical forces, with subsequent effects on all structural levels of MP physio-chemistry. MP resin pellets from the SS Hamada are auxiliary objects to investigate degradation of plastics with known age in the environment. Our study provides first data on weathering of MP from the SS Hamada. Future long-term investigations should shed light on both vaguely described mechanisms of plastic decay and the timescales in which morphological, chemical and rheological changes in MP appear.

## Methods

### Scientific diving

For sampling of MP under water, SCUBA diving following the safety standards was used. Under water, diver supported methods allow exact in situ observations, documentation, and precise sample collection. Furthermore, the standard SCUBA scientific diving methods, including underwater photo documentation (TG4, Olympus, Japan) and the distinct protocol for sampling (Stuttgart protocol) were used^55^. One sample was taken from inside the cargo hold of the wreck, which could be considered as being representative, because the MP resin pellets are constantly in flow at this sample site and, thus, homogenously distributed. After SCUBA diving, MP from the wreck were dried in the shade. Samples of MP from the beach were randomly collected at five different spots, pooled into a representative composite sample, and cleaned of sand. All samples were stored in Falcon standard tubes (50 ml). No other events of pellet loss were recorded in the Wadi el Gemal national park since 1993, and so it is secured that no other than MP resin pellets from the SS Hamada were examined.

### Polymer type

Fourier transformation infra-red (FTIR) spectra of MP resin pellets (beach: 2 particles, wreck: 3 particles) and background were recorded in transmission mode with a total of 32 scans, a resolution of 4 cm^−1^ and a wavenumber range of 4000–400 cm^-1^ (Lumos FTIR spectroscope, Bruker Optik GmbH, Ettlingen, Germany). The absorption of CO_2_ and H_2_O vapor was reduced automatically in ambient air for more clarity. OPUS 7.2 was used to acquire and analyze data and identification of polymers was performed by comparison with a library of standard spectra. The matches between this and the sample spectra were evaluated with Pearson´s r. A closer look at the spectra clearly indicated that there was no easy evidence of photo-oxidation. Therefore, the hydroxyl index (3400–3300 cm^-1^) and the carbonyl index (1810—1550 cm^-1^, and at 700 cm^-^1 to 750 cm^-^1) were considered. The carbonyl and vinyl groups are in many cases regarded as the main photo-oxidation products of PE and therefore often used as parameters to evaluate ageing of PE^56,57^. The IR-spectra of the surface measurements showed strong superposition caused by biofouling (Supplementary Fig. S1 online). In some cases, the overlaps were so strong that no absorption bands were recognizable. Thus, measurements were not only taken at the sample surface, but also from the inside of the sample after cutting the particles into halves.

### Imaging methods

#### Light microscopy

Z-stacks were taken in brightfield mode with a digital 3D light microscope to investigate MP morphology (KEYENCE VHX 7000, objective: E20; KEYENCE DEUTSCHLAND GmbH, Neu-Isenburg, Germany). For side views, MP resin pellets were vertically cut in halves with a scalpel. Panels were created using FigureJ 1.39 in FIJI ImageJ 1.53c^58,59^.

#### X-ray tomography

Microfocus computed tomography (µCT) overview scans were conducted using a high resolution µCT system (n = 130 pellets from the beach and n = 483 pellets from the wreck, respectively; v|tome|x L, GE Sensing & Inspection Technologies GmbH, Wunstorf, Germany) consisting of a microfocus X-ray tube with a maximum accelerating voltage of 240 kV and a 16-bit flat panel detector (active area 2348 × 2348 pixels at 200 µm per pixel).

The µCT scans of the single pellets from the beach were performed using a high resolution µCT-System (nanotom, GE Sensing & Inspection Technologies GmbH, Wunstorf) consisting of a nanofocus X-ray tube with a maximum accelerating voltage of 180 kV and a 12-bit flat panel detector (active area 2348 × 2348 pixels at 50 µm per pixel).

The µCT scan parameters are summarized in Supplementary Table S1 online. The so acquired 2D X-ray images were reconstructed with a special reconstruction algorithm known as Filtered Back Projection, which is integrated in the reconstruction module datos|x of the CT system manufacturer. The µCT data were visualized and analyzed with the software packages VGStudioMax 3.2 (Volume Graphics, Heidelberg, Germany) and Avizo 9.7 (Thermo Fisher Scientific, Merignac, France).

### Particle characterization

#### Particle size and particle shape

Distributions of particle size and shape descriptors were obtained from 3D reconstructed µCT data. Particle size was expressed as the diameter of a sphere with the same volume as the MP particle. Three different shape descriptors were computed that characterize all relevant shape features. Particle roundness was measured by isoperimetric shape factor *f*_*1*_

1 $$ f_{1} = \sqrt {\frac{{36\pi V^{2} }}{{A^{3} }}} $$

with *A* the area and *V* the volume of MP particles. The closer *f*_*1*_ is to one, the more MP shape equals a perfect sphere. Aspect ratio was measured by elongation. That is, the ratio between the smallest and longest caliper of a particle. Elongation is close to one for elongated objects like fibers. Flatness reaches values close to one for flat objects. For harmonized reporting, all three shape descriptors were scaled to a range between zero and one^60^.

#### Color

MP color was determined with a RAL-F2 sheet (RAL gGmbH, Germany, Bonn) as reference for both n = 100 beach and wreck MP resin pellets, respectively.

#### Mass

Also, mass n = 100 MPs was determined with an analytical balance (s.d. ≤  ± 0.0001, Sartorius analytic A 120 S, Sartorius AG, Göttingen, Germany).

### Differential scanning calorimetry

To investigate the thermal behavior of MP particles, differential scanning calorimetry (DSC) was performed (DSC 204 Phoenix, Netzsch, Selb, Germany). Ten samples of about 10 mg were weighed and sealed in aluminum DSC crucibles and placed in the DSC cell. The samples were heated from -50 °C to 170 °C at a rate of 10 °C min^-1^ under nitrogen atmosphere running two cycles for each sample. The crystallinity of the materials was calculated using a heat of fusion of 293 J g^-1^ for 100% crystalline PE.

### Gel permeation chromatography

To determine molecular distribution, the polymer was analyzed using a gel permeation chromatography (GPC). Therefore, five MP from each sample site were dissolved in 1,2,4-trichlorbenzene at 150 °C. All GPC data were recorded using a high-temperature GPC system equipped with light scattering, differential refractive index and viscometer detectors (PL-GPC 220, Agilent Technologies, Inc., Santa Clara, United States of America). The detection was done by a Multi Angle static Light Scattering detector (Wyatt Dawn Heleos, Santa Barbara, United States of America). Using this approach, absolute molecular weights were obtained. As no unexposed material could be measured, data of „Raw PE “ by ter Halle and colleagues^44^ was extracted from tables SI 8A and SI 9A and used as reference in subsequent analysis.

### Rheology

Due to weathering, the extent of long-chain branching of polymers can increase^61^. This lowers chain mobility, which results in a change of rheological properties^56,57,62^. Thus, we performed rheological characterization in shear flow with a plate − plate rheometer (Rheometrics DHR 200‐D, TA Instruments, Hüllhorst, Germany) in oscillation mode. Frequency sweeps were made at 200 °C under nitrogen atmosphere, while the gap of the plates was set to 1 mm. Dynamic mechanical experiments were carried out in a frequency range from 100 to 0.1 rad s^−1^. LDPE, a shear-thinning polymer, usually shows a Newtonian plateau at low frequencies followed by a shear-thinned drop. This behavior can be described with the Carreau-model

2 $$ { }\left| {\eta^{*} } \right| = \frac{{\eta_{0} }}{{[1 + (\lambda \cdot \omega )^{2} ]^{s} }} $$

where |η*| is complex viscosity, *η*_*0*_ is zero viscosity, *λ* is a time constant whose reciprocal approximately corresponds to the above frequency and *s* is a parameter describing the slope of the shear-thinning region. In addition, storage modulus G’ and loss modulus G’’ were recorded and loss tangent was calculated as

3 $$ \tan {\updelta } = \frac{G^{\prime\prime}}{{G^{\prime}}} $$

### Statistics

All data analysis was done with R 4.1.1 and RStudio 1.4.1717 (for information on the packages used, see Supplementary). To test for differences between sample sites, Welch’s two-tailed t tests were computed with a significance level of $\alpha$ = 0.05. The “tsum.test” function from the BSDA 1.2.0^63^ package was to assess statistically significant difference in the GPC data, as only summary statistics were provided for the reference. It was generally assumed that possible deviations from normality would not have an influence on t test performance^64^. Non-standardized effect size was expressed as the difference of means and its respective 95% confidence interval.

## Supplementary Information

Supplementary Information.

## Data Availability

The datasets generated and analyzed during the current study are available in the Open Science Framework repository, osf.io/9jxzs.
