# Supplementary material for: In situ laboratory for plastic degradation in the Red Sea
Source: Sci Rep. 2022 Jul 13;12:11956. doi: 10.1038/s41598-022-15310-7 (PMC9279475; doi:10.1038/s41598-022-15310-7)
Supplement: Supplementary file 1 — Supplementary Information. [file 41598_2022_15310_MOESM1_ESM.pdf]

In situ laboratory for plastic degradation in the Red Sea

Supplementary Material

Affiliation

Franz Brümmer[1,3,#,!], Uwe Schnepf[1,#], Julia Resch[2], Raouf Jemmali[4], Rahma Abdi[1], Hesham Mostafa Kamel[5], Christian Bonten[2], Ralph-Walter Müller[3]

1 University of Stuttgart, Institute of Biomaterials and Biomolecular Systems, Research Unit Biodiversity & Scientific Diving, Pfaffenwaldring 57, 70569 Stuttgart, Germany

2 University of Stuttgart, Institut für Kunststofftechnik IKT, Pfaffenwaldring 32, 70569 Stuttgart, Germany

3 University Stuttgart, Scientific Diving Group University Stuttgart (WiTUS), Pfaffenwaldring 57, 70569 Stuttgart, Germany

4 German Aerospace Institute (DLR), Institute for Structure and Design DE, Pfaffenwaldring 38-40, 70569 Stuttgart, Germany

5 Beluga Egypt, 32 Bahaa Eldin Elghatwary St, Semoha 12th floor apartment 1203, Alexandria, Egypt

# Both authors (alphabetically ordered) contributed equally to the study

! Corresponding author: tel. +49 711 685-65083, e-mail: franz.bruemmer@bio.uni-stuttgart.de

Statistics

Here, we provide detailed information on the R packages used for statistical analysis. The package groundhog 1.5.0[1] was used for version control of packages, and packages from GitHub sources were loaded using devtools 2.4.2[2]. We read excel files with readxl 1.3.1[3], and processed data with tidyverse 1.3.1[4]. For plotting, sciplot 1.2.0[5] functions were applied to draw bar graphs with confidence intervals and MPA 1.0.0[6] to visualize particle size distributions and frequency distributions of shape descriptors. In all diagrams, the viridis 0.6.1[7] color palette was utilized. Results of null hypothesis significance testing were tidied with broom 0.7.4[8].

Tables and figures

Supplementary Table S1 Summary of µCT settings for bulk and single particle analysis.

**Parameters**	**Bulk analysis**	**Single particle analysis**
voxel size [µm]	25	3
voltage [kV]	180	80
current [µA]	300	180
X-ray tube	microfocus tube 240 kV	nanofocus tube 180 kV
exposure time [ms]	33	800
number of projections	2000	2000
averaging/skip	3/1	10/1
rotation [°]	360	360

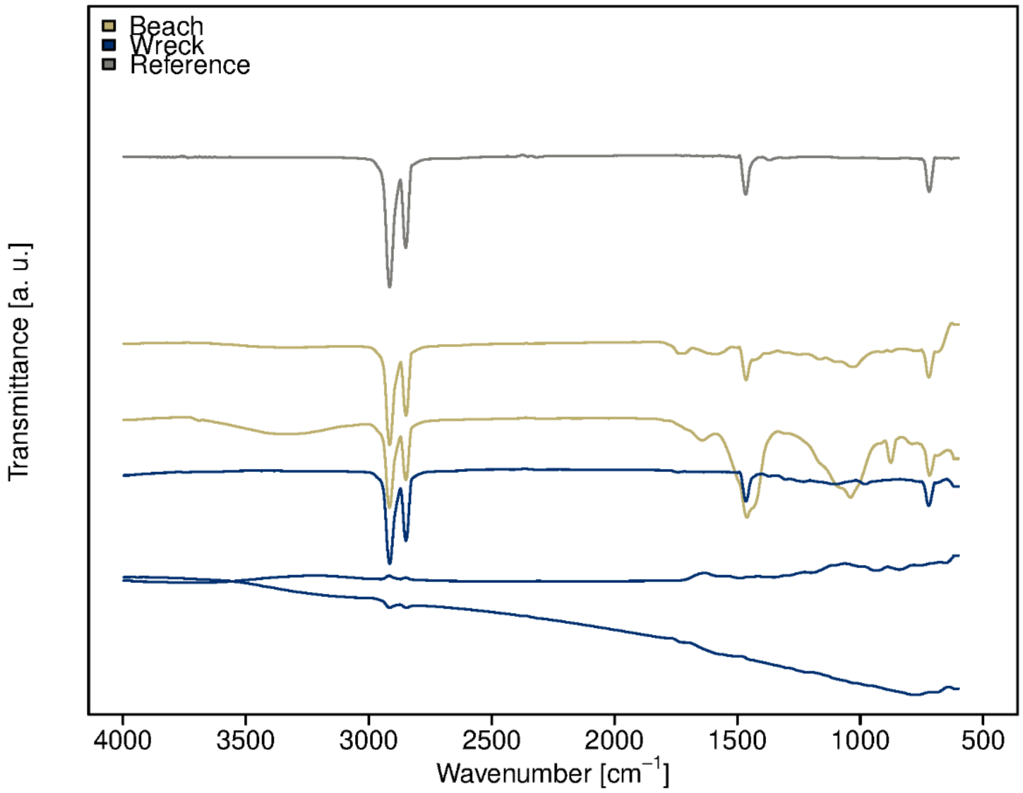

Supplementary Fig. S1 Fourier-transform infrared spectra measured on the outside of MP resin pellets from a beach in the Wadi el Gemal national park, Egypt, and the SS Hamada wreck in the Red Sea. By comparison with a reference database, all MP were identified as LDPE. Note that no meaningful spectra could have been measured for some particles as a result of biofilm formation on the surface.

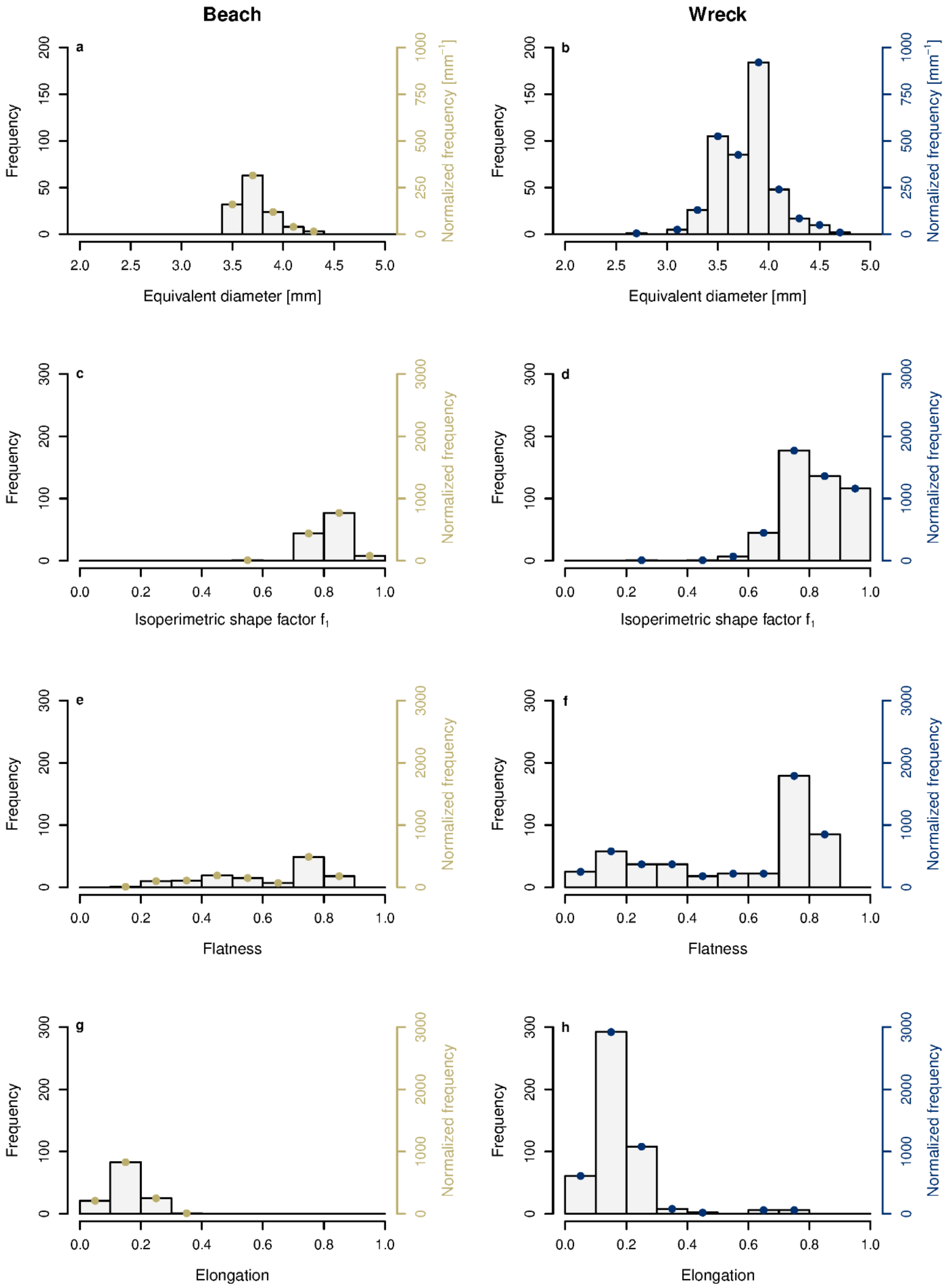

Beach
Wreck
a
b
c
d
e
f
g
h
Frequency
Normalized frequency [mm⁻¹]
Normalized frequency
Equivalent diameter [mm]
Isoperimetric shape factor f1
Flatness
Elongation

Supplementary Fig. S2 Particle size distribution and frequency distributions of three different shape descriptors of MP resin pellets from a beach in the Wadi el Gemal national park, Egypt, and the SS Hamada wreck in the Red Sea. A representative image of measured particles is shown in Supplementary Fig. S5 online. No meaningful differences between sample sites were detected. ***a*** *Particle size distribution, frequency distribution* ***c*** *of isoperimetric shape descriptor f1,* ***e*** *of flatness, and* ***g*** *of elongation of MP from the beach.* ***b*** *Particle size distribution, frequency distribution* ***d*** *of isoperimetric shape descriptor f1,* ***f*** *of flatness, and* ***h*** *of elongation for MP from the wreck. Particle size was estimated by the equivalent diameter of a sphere. All 3D shape descriptors have been normalized according to the literature[9]. Isoperimetric shape factor f_1 (equation 1) is a measure of particle roundness and approaches one for a perfect sphere. Flatness measures particle flatness with values close to one for films. Elongation describes the aspect ratio of MP with values close to one in case of fibers. Values are given for n = 130 replicates in case of MP resin pellets from the beach and n = 483 replicates in case of MP resin pellets from the wreck. Normalized frequencies were calculated according to the literature[10].*

Supplementary Table S2 Summary statistics for particle size and shape of MP resin pellets from a beach in the Wadi el Gemal national park, Egypt, and the SS Hamada wreck in the Red Sea. Size was estimated by the equivalent diameter of a sphere. All 3D shape descriptors have been normalized according to the literature[9]. Isoperimetric shape factor f_1 is a measure of particle roundness and approaches one for a perfect sphere. Flatness measures particle flatness with values close to one for films. Elongation describes the aspect ratio of MP with values close to one in case of fibers. The width of particle size distributions was determined according to standard textbooks[11].

	Size								f_1		Flatness		Elongation		N
Site	Mean [mm]	s.d. [mm]	D_{50} [mm]	IQR [mm]	D_{10} [mm]	D_{90} [mm]	D_{90}/D_{10}	Width	Mean	s.d.	Mean	s.d.	Mean	s.d.	Particle number
Beach	3.7	0.2	3.7	0.2	3.6	3.9	1.11	narrow	0.83	0.06	0.61	0.19	0.16	0.05	130
Wreck	3.8	0.3	3.8	0.3	3.4	4.1	1.19	narrow	0.82	0.10	0.56	0.27	0.18	0.10	483

s.d.: standard deviation. IQR: inter-quartile range. D_{50}, D_{10}, and D_{90} are the 50th, 10th, and 90th percentile of the particle size distribution, respectively. D_{90}/D_{10} is a ratio that describes the width of a particle size distribution[11].

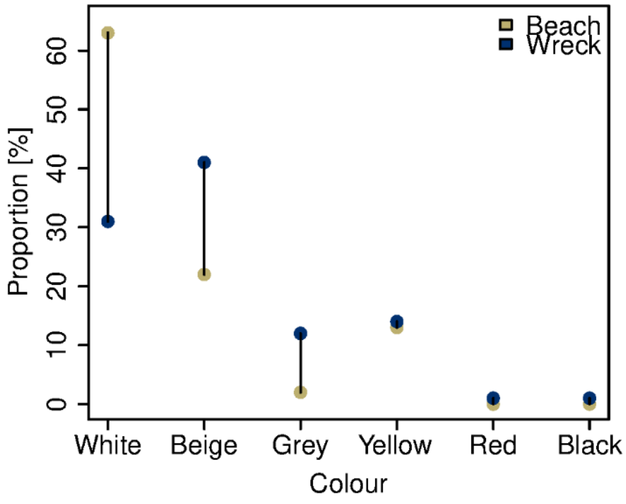

Supplementary Fig. S3 Proportion of colors of MP resin pellets from a beach in the Wadi el Gewal national park, Egypt, and the SS Hamada wreck in the Red Sea. Values are given for n = 100 replicates.

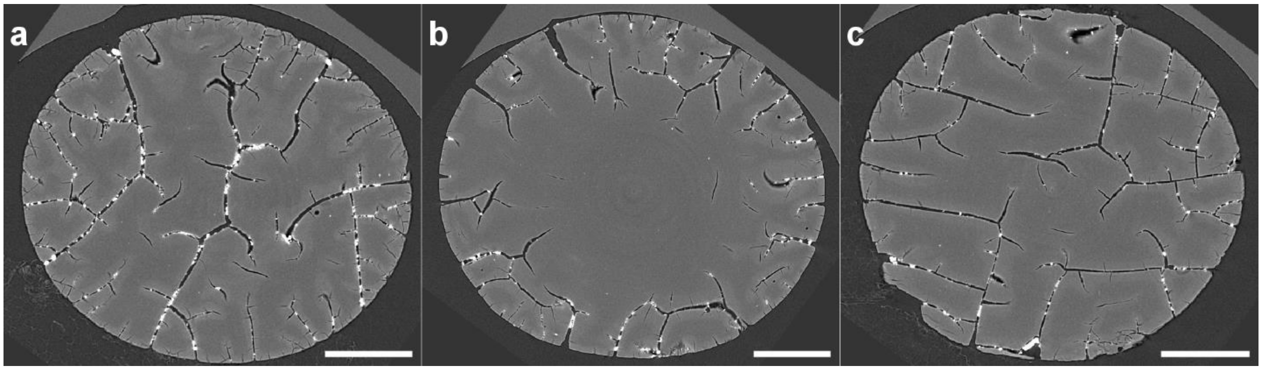

Supplementary Fig. S4 Slices of µCT scanned MP resin pellets from a beach nearby the wreck of the SS Hamada in the Wadi el Gemal national park, Egypt. Crack propagation was more pronounced near the particle outside than on the inside. ***a*** *Representative slice that is 25 %,* ***b*** *50 %, and* ***c*** *75 % distant from the upper surface of the pellet. Scale bars: 1 mm.*

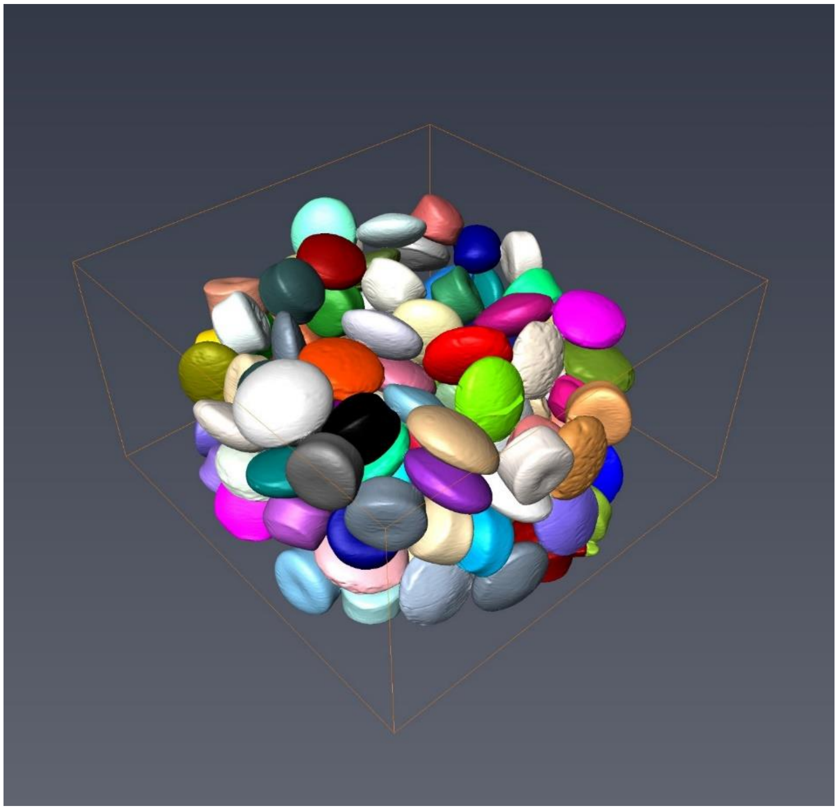

Supplementary Fig. S5 Pseudo-colored 3D reconstruction of a sample made of MP resin pellets from a beach in the Wadi el Gemal national park, Egypt.

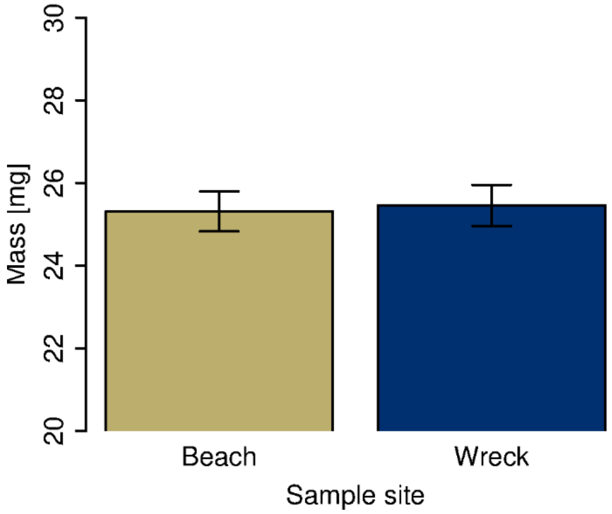

Supplementary Fig. S6 Mass of MP resin pellets from a beach in the Wadi el Gemal national park, Egypt, and the SS Hamada wreck in the Red Sea. Values are given as mean ± s.e.m. for n = 100 replicates.

Supplementary Table S3 Qualitative comparison of the physical and chemical characteristics of MP resin pellets from a beach in the Wadi el Gemal national park, Egypt, and the SS Hamada wreck in the Red Sea. "Normal" means that a certain characteristic was comparable to pristine plastic, while "higher" indicates that the respective MP particle showed characteristics that are not typical for pristine material. n. d.: not detected.

Sample site	Crack number	Surface abrasion	Molar mass	FT-IR spectra	Yellowing	Rheology
Wreck	Normal	Untypical	Untypical	Normal	Normal	Normal
Beach	Untypical	Untypical	n. d.	Untypical	Untypical	Untypical

References

1. Simonsohn, U. & Gruson, H. groundhog: Reproducible scripts via version-specific package loading. (2021).

2. Wickham, H., Hester, J. & Chang, W. devtools: Tools to Make Developing R Packages Easier. (2021).

3. Wickham, H. & Bryan, J. readxl: Read excel files. (2019).

4. Wickham, H. *et al.* Welcome to the tidyverse. *J. Open Source Softw.* **4**, 1686 (2019).

5. Morales, M. sciplot: Scientific graphing functions for factorial designs. (2020).

6. Schnepf, U. MPA: a R package for data analysis and visualization of particle measurements in microplastic research. (2021) doi:10.5281/zenodo.5027655.

7. Garnier, S. viridis: Default color maps from 'matplotlib'. (2018).

8. Robinson, D., Hayes, A. & Couch, S. broom: Convert statistical objects into tidy tibbles. (2021).

9. Crompton, C. Particle shape - An important parameter in pharmaceutical manufacturing. *Pharmaceutical Manufacturing and Packing Sourcer* (2005).

10. Filella, M. Questions of size and numbers in environmental research on microplastics: methodological and conceptual aspects. *Environ. Chem.* **12**, 527 (2015).

11. Merkus, H. G. *Particle size measurements - Fundamentals, Practice, Quality*. vol. 17 (Springer Netherlands, 2009).
